# Initial Experience With a Full Endoscopic Facet Fusion in Combination With Endoscopic Interbody Fusion

**DOI:** 10.7759/cureus.14327

**Published:** 2021-04-06

**Authors:** Jacques Lara-Reyna, Konstantinos Margetis

**Affiliations:** 1 Neurological Surgery, Mount Sinai Health System, New York, USA

**Keywords:** endoscopy, lumbar degenerative disease, minimally invasive spine, lumbar fusion

## Abstract

Background

Facet fusion has been described in open and minimally invasive approaches to promote fusion. Our objective is to describe the technique of an endoscopic facet decortication and allograft placement as an adjunct to an interbody fusion.

Methodology

This was a descriptive analysis of patients who underwent endoscopic interbody fusion combined with facet fusion and percutaneous screw placement. General demographics, clinical presentation, length of stay, follow-up, and outcome were gathered. The technique involves endoscopic access to the Kambin’s triangle, discectomy/endplate preparation, expandable cage/allograft insertion, and percutaneous pedicle screw placement. A midline incision was performed, and the endoscope was advanced over the facet joints at the desired level. After removing the soft tissue with grasping forceps, cautery was used to disrupt the facet capsule. An articulating high-speed bur was used to drill inside and over the dorsal surface of the joint. Finally, allograft chips were placed through the endoscope cannula.

Results

From May 2019 to December 2019, four patients underwent endoscopic interbody fusion. All were female, with a mean age of 67.5 years (SD: 12.7). All had chronic low back pain and radiculopathy associated with Grade 1 spondylolisthesis. Two (50%) of the patients underwent two-level fusion. The median hospital stay was two days. Two (50%) reported improvement of both low back and radiculopathy symptoms. None of the patients had a significant complication or required reoperation in eight months’ mean follow-up.

Conclusions

Facet decortication and allograft placement are feasible using an endoscopic approach in conjunction with interbody fusion.

## Introduction

Dr. Russel Hibbs was the first to describe the surgical technique for spinal fusion in Pott disease patients. Over time, and in an effort to promote fusion, he added the decortication of the facet joints to the disruption of the posterior elements [1]. Watkins et al. enhanced the Hibbs technique, using a posterolateral fusion, focusing on the grafting of the area defined by the base of the transverse process, the facet joints, and the pars articularis [2]. Nowadays, the lumbar fusion technique has evolved and can be performed in a minimally invasive (MIS) way [3].

The endoscopic lumbar fusion is one of the latest iterations of lumbar fusion techniques and carries significant potential. The technique is continually evolving. Full endoscopic is defined as surgery: (1) through a single incision, (2) under constant irrigation, and (3) visualization of the operative field through a liquid medium (irrigation fluid) and not air medium, such as in MIS cases done with tubular retractors.

The main objective of this project is to introduce and describe the concept and technical nuances of a purely endoscopic facet decortication to promote facet fusion as an adjunct to an endoscopic lumbar interbody fusion for the degenerative lumbar disease.

## Materials and methods

After obtaining approval from the Institutional Review Board (HS#20-00067. GCO#1: 20-0220 (0001) ISMMS), we performed a retrospective review of patients who underwent MIS endoscopic interbody fusion to treat the degenerative lumbar disease from May 2019 to December 2019. General demographics, associated morbidities, presenting symptoms, preoperative radiological data, length of stay, clinical/radiological outcomes, number of comorbidities, and Charlson Comorbidity Index (CCI) [4] were recorded. Descriptive analysis of the variables mentioned above was performed.

We also provide a detailed description of the technique, focused on the endoscopic facet disruption and decortication as an adjunct to an interbody fusion.

Surgical technique

The patient is positioned prone on the Jackson table. Identification of the desired incision level is obtained using lateral fluoroscopy. Preparation and draping are done in the standard sterile fashion. A #10 blade is used to incise the skin approximately 10 cm from the midline on the more symptomatic side. We then insert a needle under fluoroscopy guidance in the intervertebral disc through Kambin’s triangle at the level we intend to fuse. We remove the needle’s stylet and insert the guidewire, followed by the endoscopic system’s first dilator. Over the first dilator, we insert the endoscopic system’s cannula, which is docked in place inside the disc. Through the endoscopic cannula, we insert the endplate shavers and pituitary rongeur from the RISE® IntraLIF® system (Globus, Audubon, PA, USA). These instruments are inserted in the appropriate depth confirmed with fluoroscopy and are used to initially prepare the disc space in a percutaneous fashion. Once adequate disc resection is achieved, we insert the endoscope Elliquence® (Baldwin, NY, USA) or Richard Wolf® (Vernon Hills, IL, USA) and then use the articulating bur Richard Wolf® (Vernon Hills, IL, USA) to perform the resection of the cartilage from the endplates until bleeding bone is recognized. We then change the cannula to the cannula of the RISE® IntraLIF® system. We insert demineralized bone matrix in the disc space and then we insert the expandable cage of the RISE® IntraLIF® system, which is expanded to the desired height under fluoroscopy. The basic steps of this disc preparation technique have been demonstrated to the senior author by Dr. James J. Yue. We then proceed with the placement of the percutaneous screws CREO MIS® system (Globus Audubon, PA, USA) using the standard percutaneous technique.

**Figure 1:**
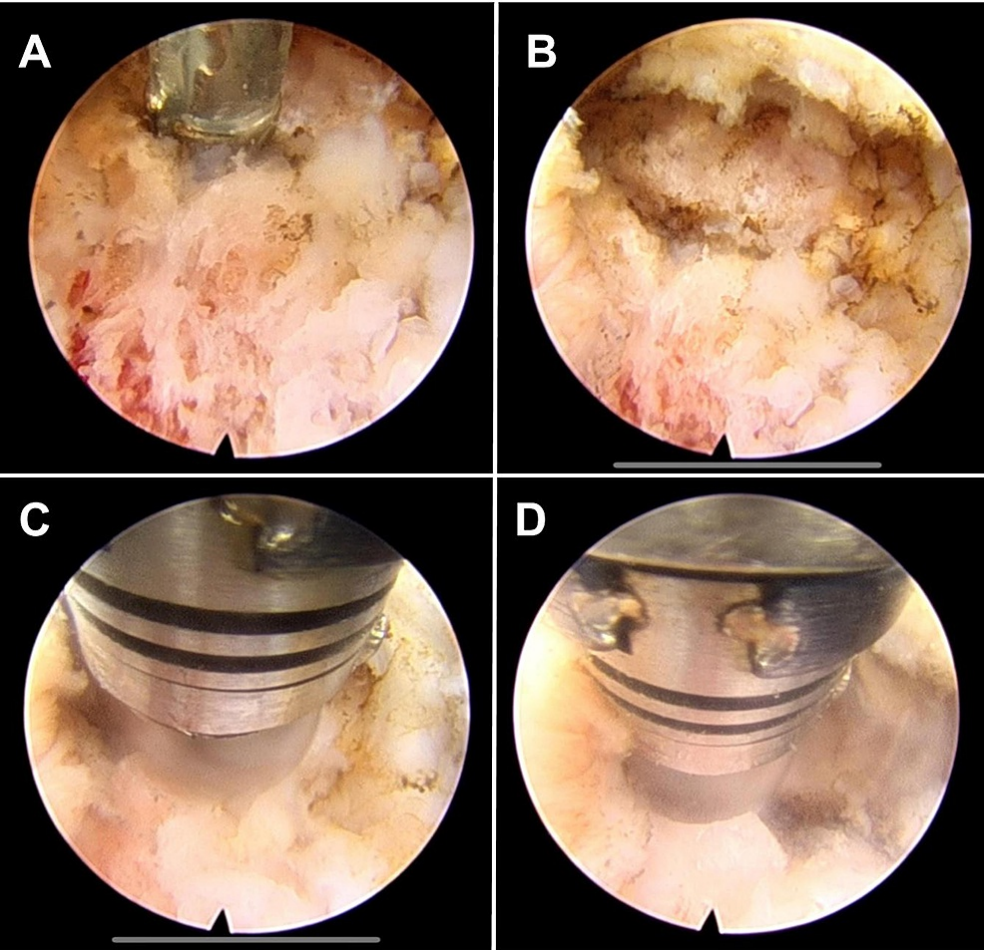
Endoscopic view of the right facet preparation. (A) Use of endoscopic bipolar to disrupt the capsule of the joint. (B) Visualization of the facet joint after removal of soft tissue. (C and D): Articulated burr is used in different directions over and within the facet joint to prepare the fusion bed before grafting.

**Figure 2:**
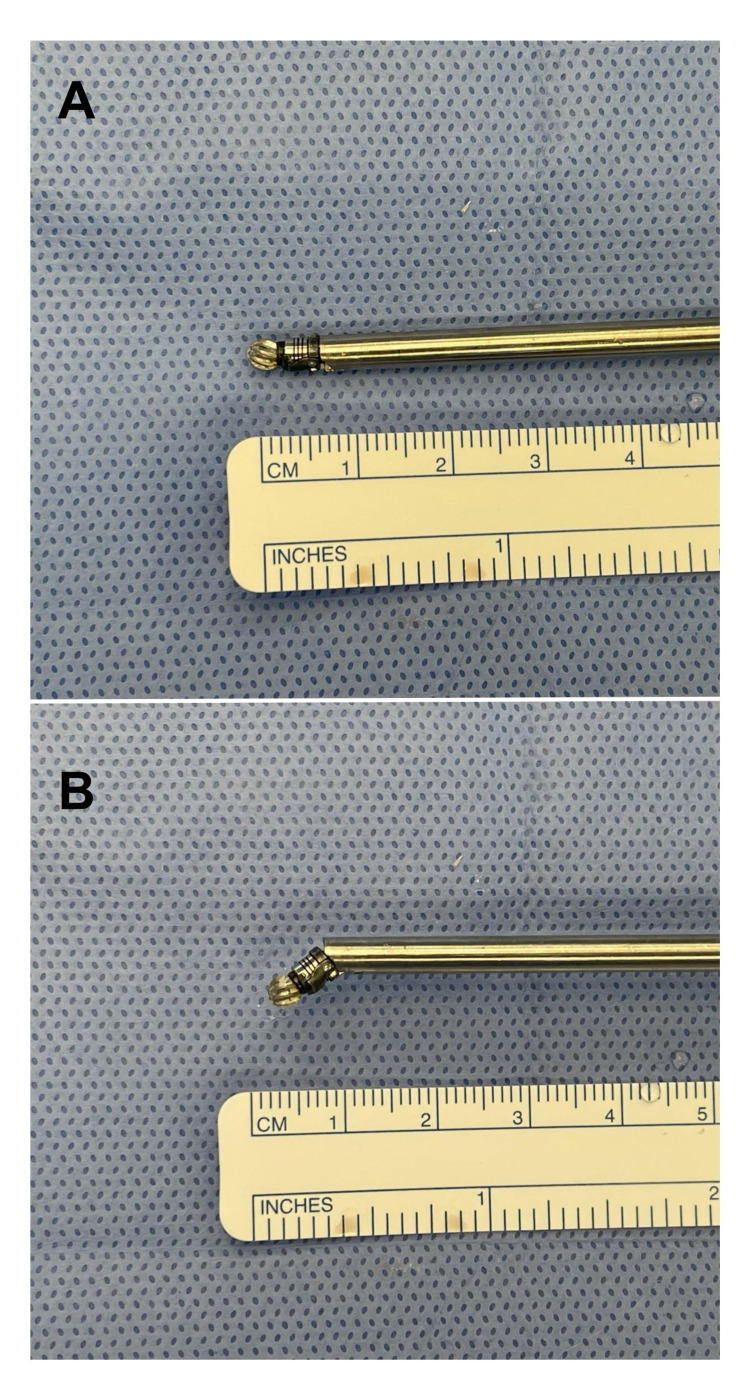
Articulating endoscopic burr. (A) In neutral. (B) In angulated position

A 7 mm midline incision is then performed, and the dilator is advanced under fluoroscopic guidance over the facet joints of the level we intend to fuse. Under endoscopic visualization, we identify the facet capsule of the target level. We remove the soft tissue over the facet joint using electrocautery and endoscopic forceps. The electrocautery Elliquence® is used to disrupt the facet capsule (Figure 1A), and the facet joint space is visualized (Figure 1B). The articulating high-speed bur is used to drill inside and on the dorsal surface of the facet joint (Figures 1C, 1D, 2).

**Figure 3:**
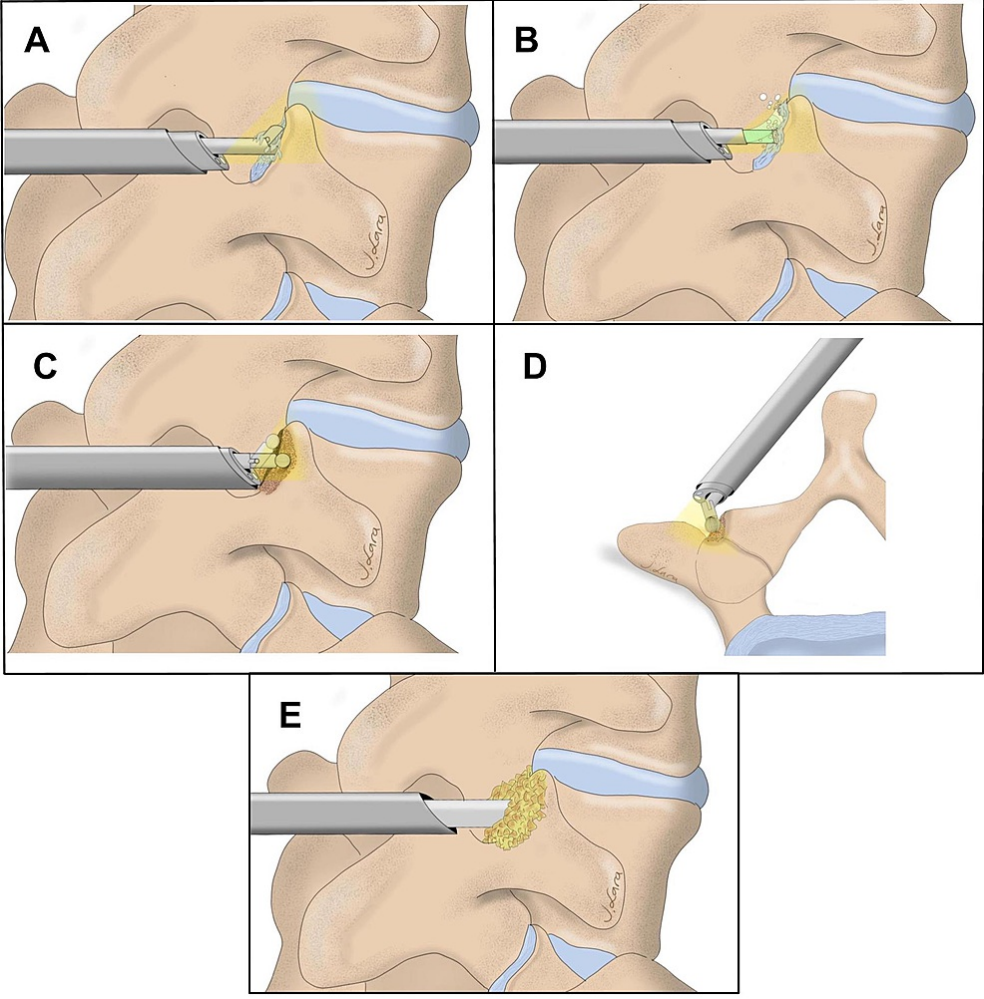
Illustration of the facet preparation and grafting using an endoscopic approach. (A) Endoscopic grasping forceps are used to remove soft tissue and the articular capsule. (B) Endoscopic bipolar is used to penetrate and disrupt the capsule. (C) (oblique view) and D (axial view): Articulating burr is used to drill and prepare the osseous and intraarticular facets before grafting. (E) Allograft is placed into and over the facet joint using the endoscopic dilator’s back end to push it through the endoscopic cannula.

We then remove the endoscope, and through the endoscopic cannula, we insert crushed allograft chips. The allograft chips are pushed into the facet joint using the back end of the endoscopic system dilator as a plunger. The same process is repeated on the contralateral side and in the additional lumbar levels in case of multi-level fusions. The purpose of this process is to achieve arthrodesis through the facet joints (Figure 3). Direct endoscopic decompression is then performed if indicated.

## Results

During the study enrollment period, four patients underwent endoscopic interbody fusion. All were female, with a mean age of 67.5 years (standard deviation [SD]: 12.7), body mass index (BMI) of 31.1 (SD: 2.74). They had a mean of 6.25 comorbidities (SD: 1.89), including cardiac, vascular, respiratory, rheumatological, and infectious diseases. A mean of 3.75 (range: 3-5) points in the CCI was obtained, which translates to a 10-year survival rate between 21% and 77%. All had chronic low back pain and radiculopathy in the setting of Grade 1 spondylolisthesis, as well as dynamic instability that had failed non-operative treatment. Two (50%) of the patients underwent two-level fusion, while the other two underwent single-level fusion. The median hospital stay was two days, while one patient had an extended hospital stay due to social issues complicating the discharge, along with residual radiculopathy. Two (50%) patients reported improvement of both low back and radiculopathy symptoms, one (25%) reported improvement in low back pain only, and one (25%) reported improvement in radiculopathy only. One patient was lost to radiological follow-up. None of the patients had a complication or required reoperation in 8 months of mean follow-up. Table 1 summarizes the clinical characteristics of the series.

**Table 1:** Case series of patients who underwent endoscopic interbody and facet fusion. LBP: low back pain; BLE: bilateral lower extremities; BMI: body mass index *Patient had an unexpected long length of stay due to social reasons

Case	Age	BMI	Symptoms	Charlson Comorbidity Index	Fused level(s)	Length of stay	Follow-up (months)	Outcome
1	72	27.21	LBP, BLE radiculopathy	4	L4-S1	*5 days	11	Improvement in LBP only
2	80	31.31	LBP, BLE radiculopathy	5	L4-L5	2 days	9	Improvement
3	50	33.25	LBP, BLE radiculopathy	3	L4-L5	1 day	11	Improvement in radiculopathy only
4	68	32.77	LBP, R radiculopathy	3	L3-L5	12 hours	15	Improvement

## Discussion

The benefits of a minimally invasive transforaminal lumbar interbody fusion (TILF) lies in reduced blood loss, lower risk for transfusion, less disruption of normal musculature, lower time to resume ambulation, and decreased time of hospitalization [5-7].

Radiological successful fusion has also been reported using MIS techniques [8,9]. Lee et al. showed that a 92.8% satisfactory fusion rate (Grade 1 and 2 in Bridwell grades) was obtained in a case series of MIS TLIF [8].

The facet joints and posterolateral structures are essential areas for fusion using decortication and grafting to promote arthrodesis. Slappey et al. demonstrated that the transverse process (especially in its base), pedicles, and lateral facets are formed of cancellous bone containing a rich vascular bed [10]. Toth et al. demonstrated satisfactory ex vivo facet fusion rate and stiffness on ovine instrumented fusion levels using recombinant human bone morphogenetic protein-2 and iliac crest graft [11]. Targeting the facets as the only fusion site has been proposed using a dedicated interfacetary grafting material. Marron et al. demonstrated the successful implementation of an allograft milled bone dowel facet fusion system to augment laminectomy and transverse process fusion procedures for the treatment of stable Grade I degenerative spondylolisthesis in a cohort of 41 patients [12]. They demonstrated that 95% of the patients had spinal stability at an average of six months post-operatively and 100% had signs of early fusion [12].

A successful facet fusion is desired to prevent screw loosening when complemented with instrumentation. Kim et al. showed that patients who underwent posterolateral or interbody fusion had a 3.5% frequency of screw loosening, in contrast to 7.1% with successful unilateral facet joint fusion/posteromedial fusion, and 0% in those patients with bilateral facet joint/posteromedial fusion [13]. With the latter in consideration, we can assume that a successful bilateral facet fusion is a protective factor against screw loosening [13].

Endoscopic TLIF technique has been previously described, with some variants including uniportal, biportal, or microendoscopic approach [7,14-18]. In our series, we preferred to use a uniportal endoscopic approach. This allows a single surgeon to have enough maneuverability to drive the endoscope and manage instruments through a single working channel.

The removal of the facet capsules under endoscopic visualization with pituitary ronguers, electrocautery, and holmium lasers has been described as a method for the treatment of facetogenic pain, however, without focusing on the facet preparation and grafting as a potential location for additional fusion [15,16,19,20]. The novelty of our technique is the feasibility of performing a facet fusion by disruption of the capsule, peripheral and intra-articular drilling, and insertion of autologous graft material in the facet joint using a purely endoscopic approach. The use of an articulating drill allows the decortication to be performed deeper inside the facet joint (Figure 1C, 1D, 3).

We used a midline approach to access the facet joints. However, this technique can also be applied through the incisions performed for the percutaneous pedicle screw placement. We selected the midline approach to avoid any limitations to the facet joint access by the instrumentation.

The limitations that we report are the limited number of cases, the lack of long-term follow-up, and the retrospective nature of the data. We did not confirm the fusion with advanced imaging, such as computed tomography, because this was not clinically indicated and was not a part of the research protocol of this observational study. The operations were performed by a single surgeon, which limits the generalizability.

## Conclusions

This was a pilot study and technical note on the full endoscopic facet decortication and graft insertion to promote facet fusion as an adjunct to an endoscopic lumbar interbody fusion. We have demonstrated that this endoscopic facet fusion is feasible and appears to be safe. The effectiveness of the technique remains to be proven in larger case series with longer follow-up times and more extensive radiological post-operative imaging.
